# To the Editors of the Medical and Physical Journal

**Published:** 1803-06-01

**Authors:** Robert Low

**Affiliations:** Member of the Royal College of Surgeons, London. Tavistock Row, Covent Garden


### *To the Editors of the Medical and Physical Journal.*

GENTLEMEN,

I Am acquainted with no means by which Medical Communications are so generally diffused as through the medium of your truly valuable Miscellany; and from the very great attention you bestow upon such information as is likely to throw any light upon the science of medicine or physics, I beg leave, in case it merits your approbation, to lay before your readers, an easy and successful method of injecting the auditory organ with metal, in order to exhibit its beautiful and intricate structure, as very few individuals in this metropolis, I believe, have been successful in their attempts of this kind. The method I adopt is as follows :—The temporal bone, if not perfectly dry, is to be exposed for some time to a degree of heat so gentle as not in the least to burn it or injure its structure, until its moisture is perfectly dissipated: the squammous portion may be broken off, taking care not to injure that part of it which forms the meatus externus, and every part of the bone except the external auditory opening is to be surrounded with Paris plaster, mixed with no more water than is just sufficient to reduce it to a paste. As soon as this coating, which should be about half or three quarters of an inch in thickness, has become perfectly set, it is at first to be exposed to a very gentle and afterwards to a clear red heat, and to remain in this state until a dense smoke ceases to issue from the external meatus, which is a criterion by which I judge of the bone being sufficiently calcinated; but this point, which is of no small importance, can only be determined by practice. The metal ought now to be melted, which should be composed of eight parts of bismuth, five of lead, and three of tin, or what is still more fusible, equal parts of zinc, bismuth, and lead, and poured while the bone is almost red hot, into the opening which has been purposely left uncovered. This metal, from its very great fusibility, retains its fluidity for a sufficient length of time, to enable it to insinuate itself compleatly into the mastoid cells, semicircular canals, and cochlea. The bone, as soon as its contents have become solid, is to be put into water, which causes the plaster of Paris (which appears to have suffered a decomposition by the fire) to fall immediately into pieces; the bone will now now admit of being detached from the metal by means of a pen knife or any sharp instrument, and if the operation has been carefully conducted, a very beautiful and perfect cast will be obtained. If the above, which is the result of a variety of experiments, proves of any service to those who are zealous in anatomical and physiological research, I shall think myself very amply repaid for the trouble I have bestowed upon the subject.

I am, &c.

ROBERT LOW,

Member of the Royal College of Surgeons, London.

*Tavistock Row, Covent Garden, May* 2, 1803.

